# Thermodynamic Studies of Complexes in Cu(II)/Uridine-5′-Diphosphoglucuronic Acid System

**DOI:** 10.3390/molecules29153695

**Published:** 2024-08-04

**Authors:** Klaudia Stachowiak, Michal Zabiszak, Jakub Grajewski, Anna Teubert, Anna Bajek, Renata Jastrzab

**Affiliations:** 1 Faculty of Chemistry, Adam Mickiewicz University in Poznan, Uniwersytetu Poznanskiego 8, 61-614 Poznan, Poland; klasta5@amu.edu.pl (K.S.); zabiszakm@amu.edu.pl (M.Z.); 2 Institute of Bioorganic Chemistry, Polish Academy of Science, Zygmunta Noskowskiego 12/14, 61-704 Poznan, Poland; ateubert@ibch.poznan.pl; 3 Faculty of Medicine, Department of Urology and Andrology, Collegium Medicum in Bydgoszcz, Nicolaus Copernicus University in Torun, Jagiellonska 13, 85-067 Bydgoszcz, Poland; a.bajek@cm.umk.pl

**Keywords:** copper (II) ions, uridine derivatives, potentiometric measurements, spectroscopic studies, biological studies

## Abstract

A binary system of uridine-5′-diphosphoglucuronic acid with copper (II) ions was studied. Potentiometric studies in aqueous solutions using computer data analysis were carried out. The pH of dominance, the overall stability constants (log*β*), and the equilibrium constants of the formation reaction (log*K_e_*) were determined for each complex compound formed in the studied system. Spectroscopic studies were carried out to determine the mode of coordination in the compounds studied. Cytotoxicity and metabolic activity tests of the compounds obtained showed an increase in the biological activity of the complexes tested against the free ligand. The current research may contribute to the knowledge of complex compounds of biomolecules found in the human body and may also contribute to the characterization of a group of complex compounds with potential anticancer properties.

## 1. Introduction

Changes in the concentration of glycans and differences in their structure on the surface of cells are important for diagnosing developing cancer or infection in the body. Compounds that regulate the level of glycans in the organism are sugar derivatives of uridine diphosphate. These derivatives are also necessary for the synthesis of glycans. Due to their properties, these compounds may constitute a new group of anticancer drugs [1,2,3,4,5,6].

An important trace element in living organisms is the copper (II) ion, which can be found in the brain, liver, or bones. Copper (II) ions are part of many enzymes, including cytochrome c oxidase, peroxidase, tyrosinase, and lysyl oxidase. Furthermore, these ions are involved in the synthesis of hemoglobin and melanin [7,8,9]. Copper (II) ion concentration disorders can cause diseases such as anemia, osteoporosis, Parkinson’s disease, hernias, Menkes syndrome, Hodgkin’s disease, leukemia, and general weakness [7,8,10]. Disorders of copper homeostasis in the brain play an important role in the pathogenesis of Alzheimer’s disease (AD). Cu^2+^ binds to β-amyloid peptides, increasing their toxicity to cells. Moreover, the binding of metal ions to the peptide accelerates its aggregation, resulting in the neuronal damage characteristic of AD [11,12].

Nucleotides and nucleosides, as the basic building units of nucleic acids, are essential for the proper functioning of the human body. Both of them participate in the synthesis of lipids and the metabolism of carbohydrates and are components of many coenzymes [13,14,15,16]. One of the representatives of pyrimidine nucleosides is uridine, which is composed of sugar ribose and uracil. It is necessary in the process of RNA and glycogen synthesis and is a precursor of uridine phosphates. Uridine and its derivatives support the proper functioning of the central nervous system. Uridine-5′-triphosphate (UTP) is used during glycogen synthesis to produce uridine-5′-diphosphate glucose (UDP-glucose) [17,18,19,20,21]. Interactions of uridine and its derivatives with metal ions are important for processes in the body. A potential coordination site in the nucleoside molecule is the endocyclic nitrogen atom N(3), which is deprotonated at higher pH values. The high basicity of this donor atom promotes the formation of connections with other biomolecules that have donor atoms with lower basicity in their structures. Additional coordination sites in nucleotide molecules are the oxygen atoms of phosphate residues [15,22].

Nucleic acid bases can exist in several different forms of tautomerism. In molecules of nucleic acid bases, keto-enol and amino-imino tautomerism can occur. The formation of tautomeric forms is related to the presence of protons capable of migration in the molecules of these organic substances. The appearance of tautomeric forms is important, e.g., in pharmacy, chemistry, and physics. In addition, the formation of different tautomeric forms can influence the structural and chemical diversity of these compounds and thus their biological function. Therefore, replacing one nuclear base with its tautomer can result in an incorrect base pair, causing an error in the genetic code or a mutation [22,23,24].

D-glucuronic acid (GluA), classified as uronic acids, contains a carboxyl, hydroxyl, and pseudo-aldehyde group in its structure and is obtained in the process of dehydrogenation of UDP-glucose [25,26,27]. These compounds play an important role in the glucuronidation process. This process allows the removal of compounds such as bilirubin, xenobiotics, and steroid hormones from the organism [25,28]. The presence of two functional groups, carboxyl and hydroxyl, in the D-glucuronic acid molecule promotes the formation of metal–ligand complex compounds. Ligands such as D-glucuronic acid can be used as masking agents for various metals, including toxic metals [27,29,30].

Uridine-5′-diphosphoglucuronic acid (UDP-GluA), which is found in the liver and kidney, plays an important role in reactions catalyzed by UDP-glucuronyltransferases (UGT), and its synthesis is catalyzed by UDP-glucose dehydrogenase. UGTs are a group of enzymes involved in the detoxification process of a living organism. These enzymes catalyze the glucuronidation of potentially toxic and carcinogenic metabolic products such as cannabinoids and such compounds as thyroxin, some bile acids, morphine, and acetaminophen [31,32].

The following article presents the results of potentiometric and spectroscopic studies on the formation of complex compounds in the system of uridine-5′-diphosphoglucuronic acid and copper (II) ions. The internal coordination sphere in the obtained complexes was also determined. Furthermore, the antitumor properties of the obtained complexes were investigated. The current research may contribute to the knowledge of complex compounds of biomolecules found in the human body and may also contribute to the characterization of a group of complex compounds with potential anticancer properties.

## 2. Results and Discussion

### 2.1. Protonation of the Ligand

**Figure 1:**
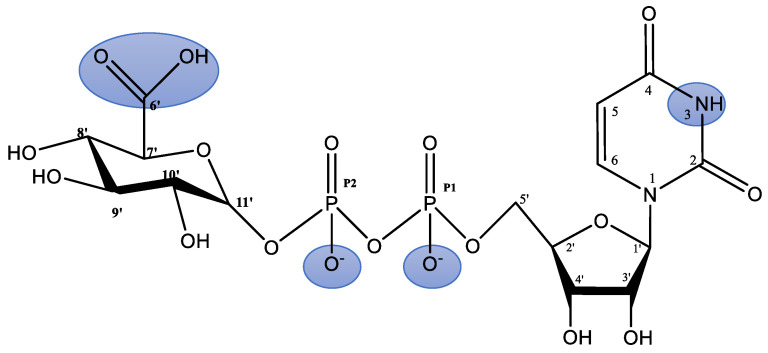
Formula of the uridine–5′-diphosphoglucuronic acid (highlighted with blue background indicate the potential coordination sites in the ligand molecule).

The analysis of the potentiometric studies confirmed the presence of three protonated forms of the ligand, (UDP-GluA)H, (UDP-GluA)H_2_, and (UDP-GluA)H_3_, in the system studied. The protonation constants of these forms and their formation reactions are given in Table 1. The structural formula of the studied ligand with a highlighted potential coordination site is shown in Figure 1.

**Table 1:** The protonation constant (log*β*) of uridine–5′-diphosphoglucuronic acid and the equilibrium constant of formation (log*K_e_*) (standard deviations are given in parentheses).

Species	log*β* [33]	log*K_e_*	Reaction
(UDP-GluA)H	8.66(2); 9.40 [33]	8.66	(UDP-GluA)^4−^ + H^+^ ⇌ (UDP-GluA)H^3−^
(UDP-GluA)H_2_	11.67(4); 12.79 [33]	3.01	(UDP-GluA)H^3−^ + H^+^ ⇌ (UDP-GluA)H_2_^2−^
(UDP-GluA)H_3_	13.85(5); 14.19 [33]	2.17	(UDP-GluA)H_2_^2−^ + H^+^ ⇌ (UDP-GluA)H_3_^−^

In the pH range studied, the carboxylic group of D-glucuronic acid, the nitrogen atom in the uridine ring, and one of the phosphate residues are deprotonated and considered potential coordination centers.

**Figure 2:**
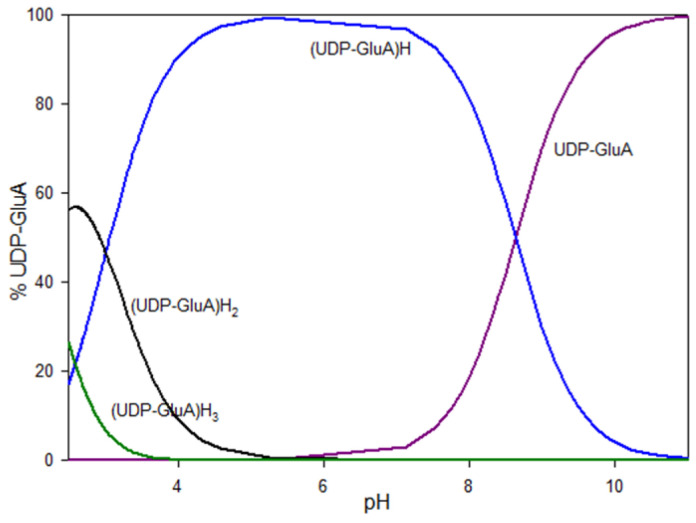
Distribution diagram of the protonation of UDP-GluA.

The deprotonation of the -O-PO_3_H^−^ group (log*β* = 13.82, log*K_e_* = 2.17) and the -COOH group (log*β* = 11.65, log*K_e_* = 3.01) starts at a pH value lower than the study range. The partially protonated form occurs in the system up to a pH value of about 5.5. The deprotonation process of the proton of -N(3)H (log*β* = 8.64, log*K_e_* = 8.64) begins below the test scale. This form is present in solution up to a pH of about 9.5 and at its maximum represents approximately 100% of all forms in the system. At a pH above 9.5, the system is dominated by the fully deprotonated form of the ligand (Figure 2).

**Figure 3:**
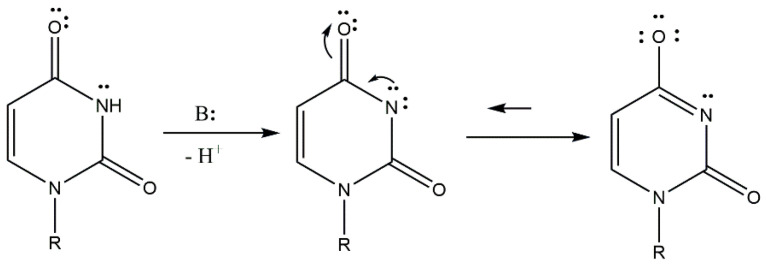
Lactam–lactim tautomerism in alkaline medium.

At high pH values, when a proton from -N(3)H is deprotonated, lactam–lactim tautomerism occurs due to the alkaline medium. As a result of tautomerism, the free electron pair from the nitrogen atom migrate, causing the formation of a double bond between the C4 carbon atom and the nitrogen atom. The formation of the double bond causes an electron pair to leave the bond between the carbon atom C4 and the oxygen atom and migrates this pair to the oxygen atom (Figure 3).

### 2.2. Binary System of Copper (II) Ion/Uridine-5′-Diphosphoglucuronic Acid

Computer analysis of the potentiometric measurements confirmed the presence of two protonated forms and two hydroxocomplexes: Cu(UDP-GluA)H_2_, Cu(UDP-GluA)H, Cu(UDP-GluA)(OH), and Cu(UDP-GluA)(OH)_3_. The stability constants (log*β*), equilibrium constants of formation (log*K_e_*), and examples of complex formation are presented in Table 2.

The correctness of the assumed model was determined by comparing the theoretical computer-generated curve and the experimental curve. The experimental and theoretical curves correspond practically over the entire range, which proves the correctness of the assumed model. The sigma parameter for the adopted model is 12.405.

**Figure 4:**
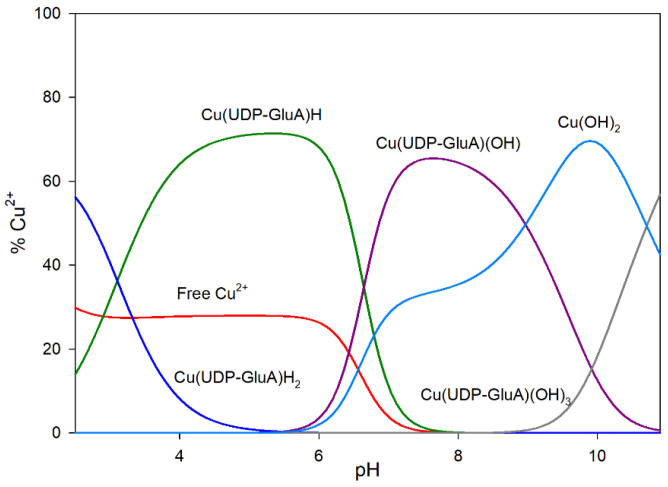
Distribution diagram of the Cu(II)/(UDP-GluA) system (ratio 1:1); $C_{{Cu}^{2+}}$ = $C_{L}$ = 1 × 10^−3^ mol/dm^3^.

Complex Cu(UDP-GluA)H_2_ starts forming at a pH below 2.5 (Figure 4). This form dominates at a pH value of approximately 2.5 and binds about 55% of the copper (II) ions. Cu(UDP-GluA)H starts forming at a pH value below 2.5. The protonated form dominates at pH 5.0–5.5 and binds maximally to 70% of copper (II) ions. The first hydroxy complex Cu(UDP-GluA)(OH) starts to form at pH 5.5 and dominates at pH values of about 8.0, where it binds approximately 65% of Cu^2+^. At pH 8.0, the last complex form starts forming: Cu(UDP-GluA)(OH)_3_. This complex dominates at a pH value above the test range. At a pH of about 10.5, this hydroxy complex binds about 55% of copper (II) ions.

The simple form of Cu(UDP-Glc) and the hydroxocomplex Cu(UDP-Glc)(OH)_2_ are most likely formed in the system under study at very low concentrations, which did not allow their identification during potentiometric tests and computer analysis.

### 2.3. Spectroscopic Studies

#### 2.3.1. UV-Vis and EPR Spectroscopy

Spectroscopic methods were used to analyze the forming complex compounds. UV-Vis and EPR measurements were performed at pH values that provided the highest percentage of a given form. These pH values were selected on the basis of distribution curves. The obtained spectroscopic parameters are summarized in Table 3. The chromophore types given in this table were determined on the basis of literature data and the results obtained and represent the possible coordination sites in the complex compounds studied.

**Figure 5:**
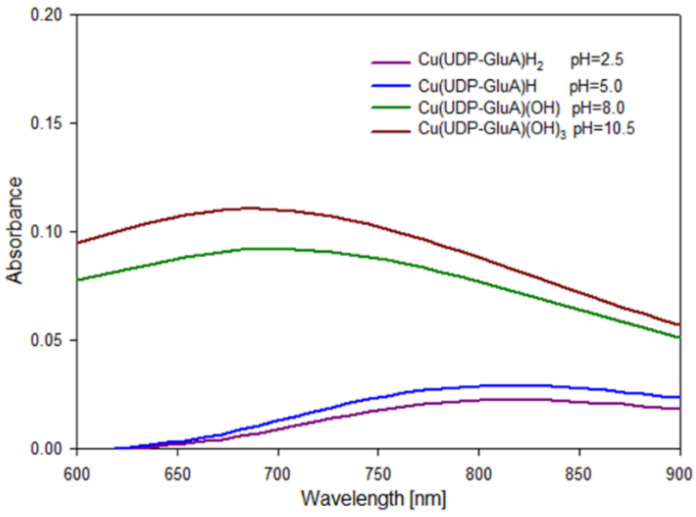
UV-Vis spectrum of the Cu(II)/(UDP-GluA) system.

The change in the internal coordination sphere in complexes containing copper (II) ions is associated with a shift in absorbance towards lower wavelengths (Figure 5). For the Cu(UDP-GluA)H_2_ and Cu(UDP-GluA)H complexes, coordination occurs through one oxygen atom derived from the glucuronic acid moiety or phosphate group. With increasing pH values, nitrogen in the UDP-GluA molecule is deprotonated and a coordination bond is formed with this donor atom. For the Cu(UDP-GluA)(OH) complex, coordination occurs through a nitrogen atom derived from the UDP molecule and two oxygen atoms derived from the glucuronic acid molecule and phosphate group of the UDP molecule. The internal coordination sphere of the last complex form from the studied system includes a nitrogen atom N(3) derived from the UDP molecule and three oxygen atoms derived from carbonyl group -COOH of glucuronic acid and two phosphate residues -O-PO_3_H.

**Figure 6:**
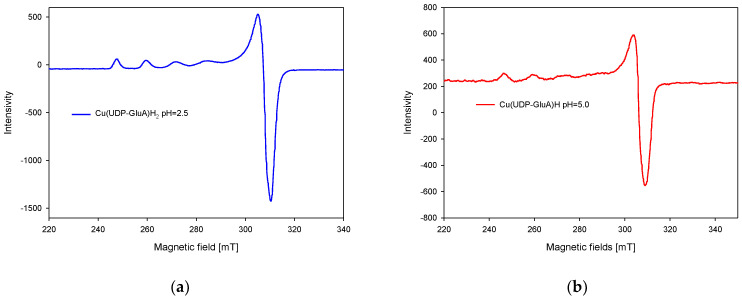
EPR spectra of (**a**) Cu(UDP-GluA)H_2_, (**b**) Cu(UDP-GluA)H.

On the basis of EPR spectra analysis, the formation of monomeric complex forms in the studied system was confirmed, and characteristic spectra for copper (II) ions were observed (Figure 6). The spectral parameters g_ǁ_ = 2.39 and A_ǁ_ = 136 × 10^−4^ cm^−1^ for the Cu(UDP-GluA)H_2_ complex and g_ǁ_ = 2.37 and A_ǁ_ = 145 × 10^−4^ cm^−1^ for the Cu(UDP-GluA)H complex indicate the participation of one oxygen atom in the formation of the coordination bond. The EPR spectra obtained are analogous due to the similar coordination mode in the compounds analyzed.

Tests were not carried out for forms formed at pH = 8.0 and pH = 10.5, due to sediment formation in the prepared samples.

#### 2.3.2. CD Spectroscopy

**Figure 7:**
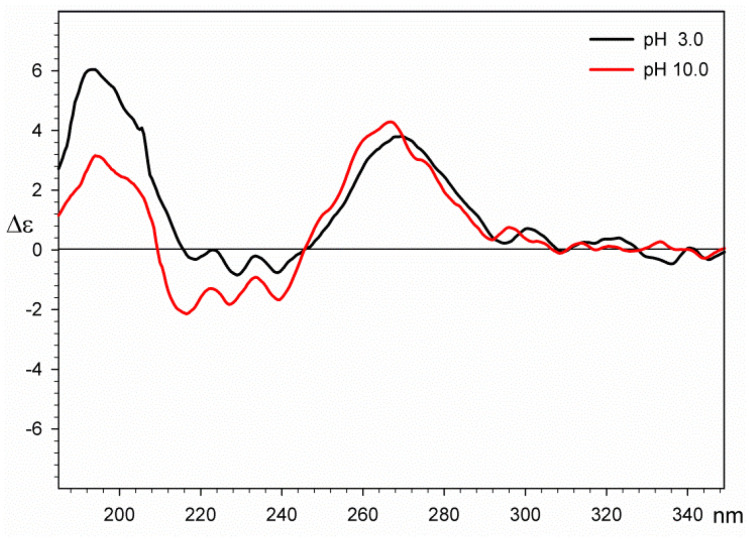
Circular dichroism spectra of uridine–5′-diphosphoglucuronic acid in water at pH = 3 (black line) and pH = 10.0 (red line).

The first series of CD measurements was carried on uridine-5′-diphosphoglucuronic acid at different pH values to exclude protonation/deprotonation effects on the conformation of the ligand. Based on the results of potentiometric studies, pH 3.0 and 10.0 were chosen. The results of these measurements show that there is no significant change in CD spectra in the range of 250–280 nm, which originates from the absorption of the uridine base. The maximum observed for the acidic solution is Δε = 3.81 at 269 nm and Δε = 4.29 at 266 for the basic one. In the short-wave part of the spectra, a negative Cotton effect can be observed for the sample in the basic solution. This result is usually related [34,35] to the deprotonation of chiral carboxylic acid, which shifts the n-π* transition and CD maxima towards shorter wavelengths (Δε = −0.84 at 228 nm for the acidic solution nm and Δε = −2.14 at 216 nm for the basic one). The loaded CD spectra for both solutions are presented in Figure 7 and indicate that the conformation of uridine-5′-diphosphoglucuronic acid is not pH-dependent in water solutions.

As the uridine-5′-diphosphoglucuronic acid has many potential electron donor sites, the possible coordination of Cu(II) ions may take place by different parts of the ligand cause its conformational changes. These coordination modes may also vary due to pH changes. The CD measurements of the Cu(II)/UDP-GluA system were performed at a pH that was previously determined on the basis of potentiometric measurements knowing that the main form of the complex is usually present alongside other minor ones. In the case of measurements for all pH, the same sequence of Cotton effects is observed with a positive long-wave effect located in the range of 262–272 nm and negative Cotton effects located in the range of 218–239 nm. The main Cotton effects at a given wavelength are summarized in Table 4.

**Figure 8:**
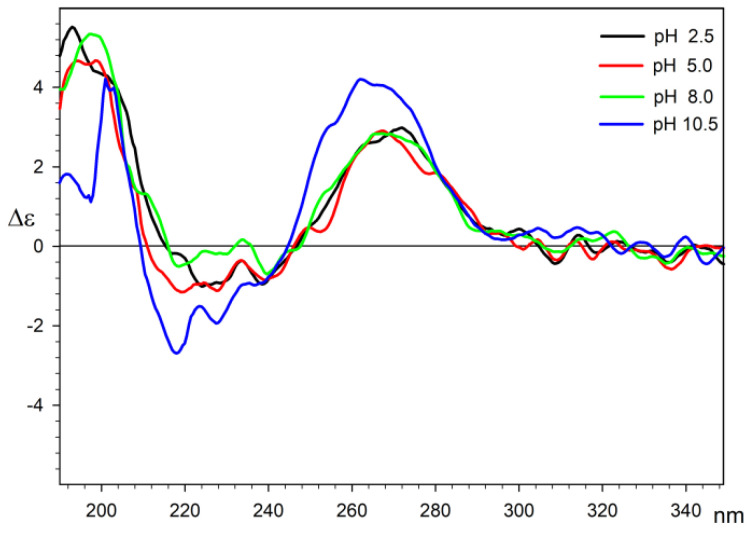
Circular dichroism spectra of the Cu(II) uridine–5′-diphosphoglucuronic acid system in water at pH = 2.5 (black line), pH = 5.0 (red line), pH = 8.0 (green line), and pH = 10.5 (blue line).

The results indicate no substantial changes in the ligand conformation in the pH range of 2.5–8.0. The spectrum measured at pH = 10.5 has a more intense and blue-shifted maximum, which is in agreement with NMR shift measurements and may be associated with the change in donor atoms in the system. Additionally, this change can also be connected with the shift in equilibrium of base-promoted lactam–lactim tautomerization, which is also observed in the shift in the C4 signal in the ^13^C NMR spectra. The negative Cotton effects in the range of 218–239 nm are derived from the interaction of the carboxylic acid residue with the chiral environment. As expected, there is a general tendency to increase the intensity of these effects and shift towards shorter wavelengths with increasing pH; however, the mutual proximity of positive Cotton effects of relatively high intensity may affect their height and location on the CD spectrum, which makes their precise interpretation difficult. CD spectra of the Cu(II)/UDP-GluA system are presented in Figure 8.

#### 2.3.3. NMR Spectroscopy

NMR studies were conducted to determine the mode of coordination in the studied system. The studies were performed for ligands and complexes for two pH values. The results obtained are summarized in Table 5.

**Figure 9:**
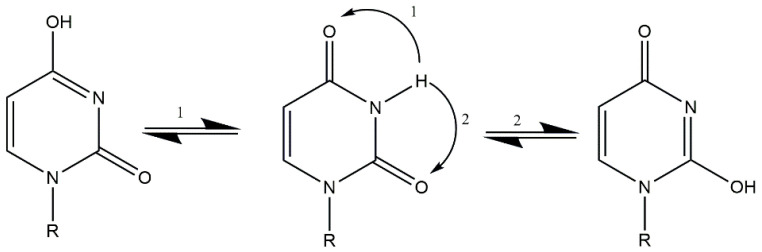
Lactam–lactim tautomerism in the uridine moiety and its derivatives [22].

Analysis of ^13^C NMR and ^31^P NMR spectra revealed changes in the chemical shifts between the free ligand and the complex compound. At low pH values, changes in chemical shifts were observed in carbon C6′ (0.20) derived from the glucuronic acid moiety and at the phosphorus atom P2 (7.93). At these pH values, coordination occurs between the oxygen atom of glucuronic acid and the oxygen atom of the phosphate group of the UDP moiety. This type of coordination occurs for the complex Cu(UDP-GluA)H. At higher pH values, changes in chemical shifts were observed at the C4 (−0.91) carbon atom and P2 (4.5) phosphorus atom of the UDP molecule and the C6′ (−0.70) carbon atom of the glucuronic acid moiety. These changes are due to deprotonation of the nitrogen atom at higher pH values and the occurrence of lactam–lactim tautomerism. These changes explain the chemical shift in only the C4 carbon and not the C4 and C2 carbons in the ^13^C NMR spectrum. At pH 8.0, coordination occurs through an oxygen atom located at the C4 carbon in the uridine ring and through one of the oxygen atoms of the phosphate group and an oxygen atom derived from the carbonyl group of D-glucuronic acid. A schematic of the lactam–lactim tautomerism is shown in Figure 9.

#### 2.3.4. Cytotoxicity and Metabolic Activity Tests

**Figure 10:**
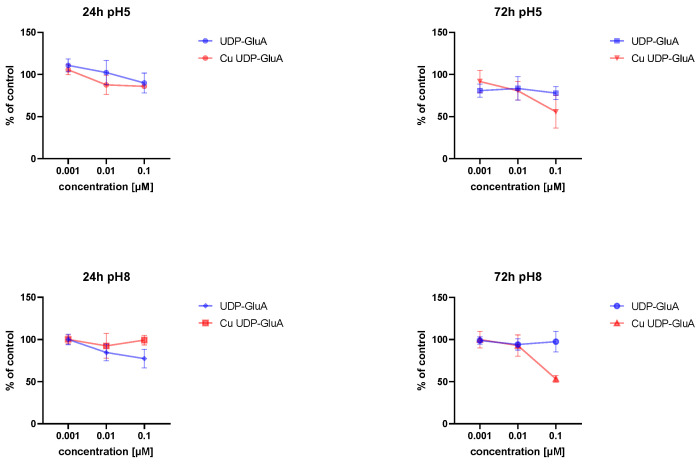
Cell viability after incubation with tested compounds for 24 h and 72 h in different pHs.

The IC50 (half maximal inhibitory concentration) is defined as the concentration of compound needed to inhibit a biological process or response by 50%. The metabolic activity of the cells after 24 h of incubation with tested compounds at pH 5 was similar (Figure 10). The same was true for IC50 values (Table 6). However, after 72 h, a higher cytotoxicity could be observed for Cu UDP-GluA. A549 cells reduced their activity by 44.4% in comparison to the control, e.g., cells cultured in standard growth medium.

In turn, after 72 h of incubation with UDP-GluA at pH 8, no changes in the cells’ metabolic activity could be detected; therefore, the IC50 value could not be calculated. Cu(UDP-GluA) at pH 8 after 72 h of incubation reduced the A459 cell’s metabolic activity by 52.2%. However, the effect was strongly time-dependent (Table 6).

## 3. Materials and Methods

### 3.1. Materials

Uridine-5′-diphosphoglucuronic acid trisodium salt was obtained from Sigma-Aldrich and copper (II) nitrate was obtained from Merck (Sigma-Aldrich Merck group, Burlington, MA, USA). All of these materials were used without purification.

### 3.2. Potentiometric Studies

Potentiometric titration was performed using a Titrando 713 Methrom equipped with an autoburette-combined glass electrode calibrated prior to each test (Methrom, Herisau, Switzerland). Calibration was performed prior to each titration with two buffer solutions of pH 4.002 and pH 9.225. All measurements were carried out under strictly defined conditions of a constant ionic strength of 0.1 M KNO_3_, temperature of 20 ± 1 °C, an inert gas atmosphere of helium (He 5.0 Ultra-High Purity), and a pH range of 2.5 to 11.0. The metal-to-ligand ratio was 1:1 and the concentration of ligands and copper (II) ions [Cu^2+^] was 0.001 mol/dm^3^. The protonation constant and the stability constant of the complex were determined using the HYPERQUAD 2008 program. The calculations allowed us to determine the model of complex formation in the systems studied. The correctness of the assumed model was verified by analyzing the standard deviations and the convergence of the experimental and theoretical curves [36].

### 3.3. UV-Vis Spectroscopy

UV-Vis spectroscopy studies were performed on a SHIMADZU UV-1900 spectrophotometer using the UVProbe program (SHIMADZU, Kyoto, Japan). Measurements were made in the wavelength range of 550 to 900 nm. The concentration of the metal ion was 0.001 mol/dm^3^ and the metal-to-ligand molar ratios was 1:1. The data obtained with UVProbe were saved as a text file, and the spectra were produced with SigmaPlot 11.0.

### 3.4. EPR Spectroscopy

The EPR spectra were carried out at a temperature of −196 °C, using glass capillary tubes, and recorded on an SE/X2457 Radiopan spectrometer. EPR studies were performed for copper (II) ion systems, in which the concentration of metal ions was 0.005 M in a solution of water/glycol of 3:1 with a metal–ligand ratio of 1:1.

### 3.5. NMR Spectroscopy

^13^C and ^31^P NMR spectroscopic measurements were recorded using a 400 MHz (9.39 T) AVANCE II Bruker NMR spectrometer (Bruker, Billerica, MA, USA). Investigations were performed for the ligand and complex forms formed at two pH values: pH = 5.0 and pH = 8.0. Samples were prepared by dissolving the corresponding reactants in D_2_O and the pD values of each prepared sample were determined by NaOD and DCl taking into account the relation pD = pH + 0.4 [37]. The concentration of ligands in the samples was 0.05 mol/dm^3^ and the M:L ratio was 1:100.

### 3.6. CD Spectroscopy

The CD and corresponding UV spectra were recorded on a JASCO J810 spectropolarimeter at ambient temperature (JASCO, Halifax, NS, Canada). Spectra were recorded in the range of 185–400 nm in water solutions and accumulated with four scans for both UDP-GluA and its copper (II) complexes. Water for the experiments was extra-purified by the Merck Millipore Simplicity UV apparatus to lower the absorbance especially in the short-wave part of the measuring range. The measurements were performed in N_2_ gas atmosphere (flow 10 L/min) and the optical path length was 0.1 cm [38]. The concentrations of measured solutions were 1 × 10^−4^ M, which allowed the absorbance to be maintained at an acceptable level.

### 3.7. Cell Line and Cell Culture

The A549 cell line was initially initiated through an explant culture of lung carcinoma tissue from a 58-year-old male. Cells were hypotriploid with a modal chromosome number of 66 in 24% of cells. As verified at ATCC, there were six markers present in single copies in all cells, e.g., der(6)t(1;6) (q11;q27), del(6) (p23), del(11) (q21), del(2) (q11), M4, and M5. Most cells have doubled sex chromosomes, single copies of N2 and N6 chromosomes, and four copies of N12 and N17 chromosomes.

Basic cell culture reagents and other materials were purchased from Corning. A549 cells were routinely cultured in F-12K medium supplemented with fetal bovine serum to a final concentration of 10%. They were maintained at 37 °C in a humidified cell culture incubator (Thermo Fisher Scientific, Waltham, MA, USA) with 5% CO_2_ at a concentration between 6 × 10^3^ and 6 × 10^4^ cells per cm^2^. The standard growth medium was changed two or three times a week.

### 3.8. MTT Assay

The assay allows for the detection of the cells’ metabolic activity. It is based on the intracellular reduction in the water-soluble MTT reagent to an insoluble formazan by respiring cells. A549 cells were detached from the growth surface, counted, and transferred to 96-well plates at a density of 4.5 × 10^3^ cells per cm^2^. After 24 h of preincubation in standard conditions, tested compounds were added, and cells were cultured for 24 or 72 h. The medium was discarded and cells were incubated with the MTT solution (1 mg/mL) for 1 h at 37 °C. Formed purple formazan crystals were dissolved in DMSO (StanLab, Lublin, Poland). The absorbance was read at OD 570 nm using a microplate reader Multiskan Sky (Thermo Fisher Scientific).

## 4. Conclusions

The formation of complexes in the binary system of uridine-5′-diphosphoglucuronic acid and copper (II) ions was established. The existence of two types of complex forms in the system was observed: MHxL and ML(OH)x. Depending on the pH, different coordination modes were observed in the obtained complex compounds. The types of chromophores were determined by UV-Vis, EPR, and NMR spectroscopic studies. It was observed that, at low pH values, coordination occurs through an oxygen atom derived from the glucuronic acid molecule or phosphate group. As the pH increases, the nitrogen atom in the ligand molecule is deprotonated and a coordination bond is formed with this donor atom. Oxygen atoms derived from UDP phosphate residues also play an important role in the coordination process. The bioassays carried out showed an increase in the biological activity of the complex compounds tested against the free ligand. As the incubation time increases, the biological activity of the tested compounds increases.

## Figures and Tables

**Table 2:** The overall stability constants (log*β*) and the equilibrium constants of formation (log*K_e_*) of the complexes formed in the studied system (standard deviations are given in parentheses).

Species	log*β*	log*K_e_*	Reaction
Cu(UDP-GluA)H_2_	15.61(3)	3.94	Cu^2+^ + (UDP-GluA)H_2_ ⇌ Cu(UDP-GluA)H_2_
Cu(UDP-GluA)H	12.52(2)	3.86	Cu^2+^ + (UDP-GluA)H ⇌ Cu(UDP-GluA)H
Cu(UDP-GluA)(OH)	−0.78(2)	12.98	Cu^2+^ + (UDP-GluA) + H_2_O ⇌ Cu(UDP-GluA)(OH) + H^+^
Cu(UDP-GluA)(OH)_3_	−20.87(3)	7.45	Cu(UDP-GluA)(OH) + 2H_2_O ⇌ Cu(UDP-GluA)(OH)_3_ + 2H^+^

**Table 3:** UV-Vis and EPR spectroscopic parameters for the formation of complex forms.

Species	pH	g_ǁ_	A_ǁ_ [cm^−1^]	λ_max_ [nm]	ε [M^−1^cm^1^]	Abs.	Chromophore	Proposed Mode of Coordination
Cu(UDP-GluA)H_2_	2.5	2.39	136 × 10^−4^	810	23	0.023	{1O}	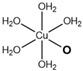
Cu(UDP-GluA)H	5.0	2.37	145 × 10^−4^	800	30	0.030	{1O}	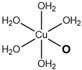
Cu(UDP-GluA)(OH)	8.0	-	-	710	93	0.093	{1N, 2O}	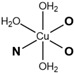
Cu(UDP-GluA)(OH)_3_	10.5	-	-	690	112	0.112	{1N, 3O}	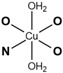

**Table 4:** Cotton effects for Cu(II)/UDP-GluA system in water solutions.

pH	2.5	5.0	8.0	10.5
Δε (nm)	2.98 (272)	2.91 (267)	2.85 (267)	4.21 (262)
	−0.95 (239)	−0.85 (239)	−0.70 (239)	−0.98 (237)
	−1.01 (224)	−1.11 (228)	−0.19 (227)	−1.94 (227)
		−1.15 (219)	−0.50 (219)	−2.70 (218)

**Table 5:** Differences between ^13^C NMR and ^31^P NMR chemical shifts from the ligand in the Cu(UDP-GluA) system in relation to the free ligand [ppm].

System	pH	(UDP-GluA)				
		C2	C4	C6′	P1	P2
Cu(UDP-GluA)H	5.0	0.06	−0.03	0.20	-	7.93
Cu(UDP-GluA)(OH)	8.0	−0.05	−0.91	0.07	0.16	4.50

**Table 6:** IC50 values [uM] calculated based on the MTT assay results.

Species	24 h	72 h
(UDP-GluA) pH5	0.317 ± 0.006	0.781 ± 0.005
Cu(UDP-GluA) pH5	0.377 ± 0.005	0.116 ± 0.005
(UDP-GluA) pH8	0.295 ± 0.004	n.d. *
Cu(UDP-GluA) pH8	1.719 ± 0.01	0.094 ± 0.004

* n.d.—not detectable, too low a cytotoxicity to calculate IC50.

## Data Availability

All data generated or analyzed during this study are included in this published article.

## Abbreviations

UDP-GluA, Uridine-5′-diphosphoglucuronic acid.
